# The effects of NaCl concentration and pH on the stability of hyperthermophilic protein Ssh10b

**DOI:** 10.1186/1471-2091-8-28

**Published:** 2007-12-21

**Authors:** Yong-Jin Mao, Xiang-Rong Sheng, Xian-Ming Pan

**Affiliations:** 1 National Laboratory of Biomacromolecules, Institute of Biophysics, Chinese Academy of Sciences, Beijing, 100101, China; 2 Department of Biological Sciences and Biotechnology, Tsinghua University, Beijing, 100084, China; 3 Graduate University of Chinese Academy of Sciences, Beijing, 100049, China

## Abstract

**Background:**

Hyperthermophiles constitute a group of microorganisms with an optimum growth temperature of between 80°C and 100°C. Although the molecular underpinnings of protein thermostabilization have been the focus of many theoretical and experimental efforts, the properties leading to the higher denaturation temperature of hyperthermophilic proteins are still controversial. Among the large number of factors identified as responsible for the thermostability of hyperthermophilic proteins, the electrostatic interactions are thought to be a universally important factor.

**Results:**

In this study, we report the effects of pH and salt concentration on the urea-induced denaturation of the protein Ssh10b from a hyperthermophile in low ionic strength buffer. In the absence of NaCl, the unfolding ΔG of the protein increased from about 33 kJ/mol at pH 3 to about 78 kJ/mol at pH 10. At all values of pH, the ΔG increased with increasing NaCl concentration, indicating that salt stabilizes the protein significantly.

**Conclusion:**

These findings suggests that the increased number of charged residues and ion pairs in the protein Ssh10b from hyperthermophiles does not contribute to the stabilization of the folded protein, but may play a role in determining the denatured state ensemble and also in increasing the denaturation temperature.

## Background

Hyperthermophiles constitute a group of microorganisms with an optimum growth temperature of between 80°C and 100°C. Proteins isolated to date are composed of the common 20 amino acids. Furthermore, homologous proteins from hyperthermophiles and mesophiles typically show 40–85% sequence similarity and their three-dimensional structures are superposable [1-4], suggesting that the factors underlying the extreme thermal tolerance are hidden in the delicate balance of the non-covalent interactions.

Although the molecular underpinnings of protein thermostabilization have been the focus of many theoretical and experimental efforts, the properties leading to the higher denaturation temperature of proteins from hyperthermophiles are still controversial [5]. Among the large number of factors identified as responsible for the thermostability of proteins from hyperthermophiles, electrostatic interactions have been proposed to be a universally important factor. A comparison of 13 structural parameters calculated from the tertiary structures of 64 proteins from mesophiles and 29 proteins from thermophiles and hyperthermophiles indicated that increases in the numbers of charged residues and ion pairs with increasing growth temperature and other parameters show just such a trend [6]. A theoretical analysis of charge interactions calculated using the Tanford-Kirkwood model applied to the protein crystal structures also demonstrated a correlation between electrostatic interactions and thermostability [7]. However, estimates of the energy contribution of an ion pair to protein stability have led to conflicting conclusions, ranging from stabilizing, through insignificant, to destabilizing effects [8-10].

Estimates of the energy contributions of ion pairs and net charges to protein stability could also be achieved by changing environmental variables such as pH and salt concentration [11]. The stability of the *Pyrococcus furiosus *methyl aminopeptidase decreased at low pH, where the acidic residues are protonated and favorable ionic interactions are disrupted, as well as at high salt concentration, where high ionic strength is known to destabilize ion pairs [12]. It was also found that NaCl destabilizes *Sulfolobus solfataricus *carboxypeptidase at pH 7.5, but not at pH 9.0, where the stabilizing ion pairs probably do not exist any more. These and other similar experiments were considered to support the suggestion that ion pairs are an essential factor contributing to the thermostability of proteins from thermophiles and hyperthermophiles [13].

The DNA binding protein Ssh10b, from the archaeon *Sulfolobus shibattae *with an optimal growth temperature of 95°C, is a member of the Sac10b family and is conserved in most thermophilic and hyperthermophilic archaeal genomes that have been sequenced to date. The protein constitutes about 4–5% of the cellular protein, binds dsDNA without apparent sequence specificity, and is capable of constraining negative DNA supercoils in a temperature-dependent fashion. This binding ability is weak at 25°C, but is enhanced substantially at 45°C and higher temperatures. Ssh10b is a dimeric protein composed of two identical subunits. The monomer of Ssh10b consists of 97 amino acid residues with no disulfide bonds and is highly thermostable [14]. The crystal structure of Ssh10b revealed a mixed α/β structure comprising four β-strands and two α-helixes [15]. In a previous study, we found that Ssh10b is resistant to urea-induced denaturation in phosphate buffer (20 mM Na_2_HPO_4_/NaH_2_PO_4_) [16]. In the present study, we report the effects of pH and salt concentration on the urea-induced denaturation of Ssh10b in monovalent ion buffer.

## Results

### pH- and salt-dependent unfolding by urea

**Figure 1:**
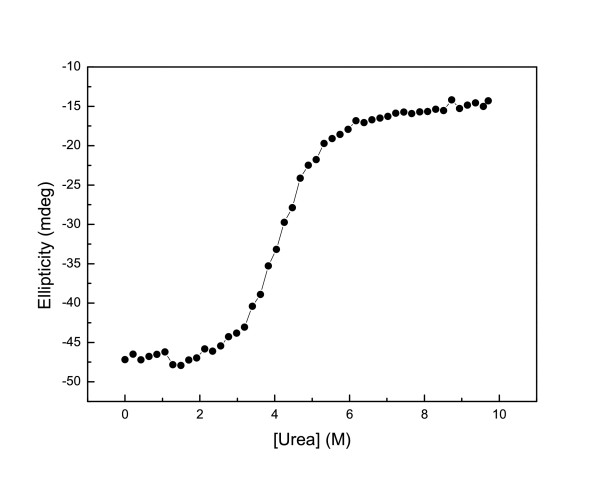
**The urea induced unfolding curve**. Unfolding at 25°C. The concentration of Ssh10b is 50 μM at pH 7 in 10 mM HEPES buffer and in the absence of NaCl.

**Figure 2:**
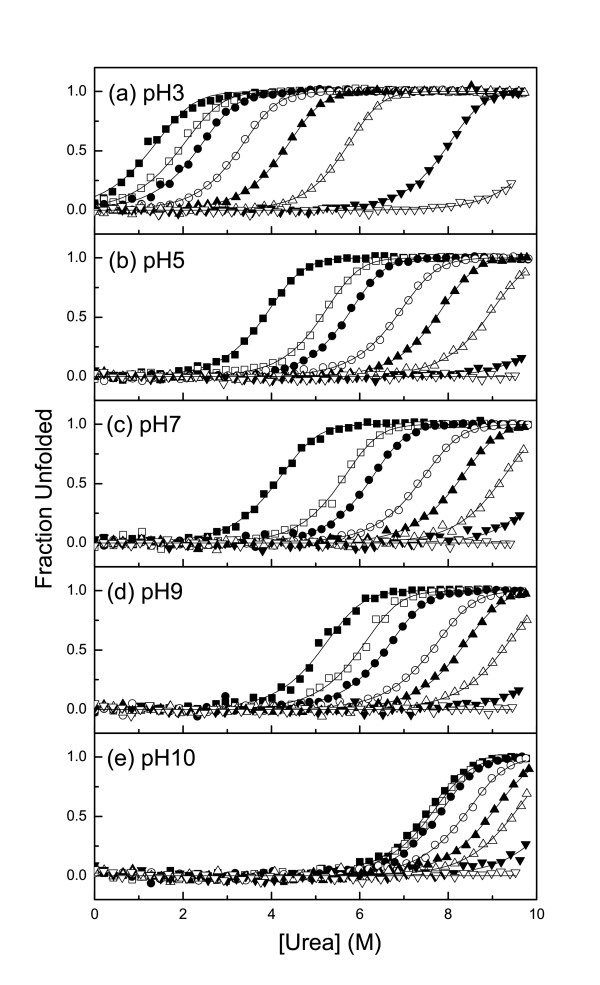
**The effects of pH and salt concentration on the urea-induced stability of Ssh10b measured at 25°C**. (a) pH = 3 (buffer: 10 mM Gly), (b) pH = 5 (buffer: 10 mM HAc), (c) pH = 7 (buffer: 10 mM HEPES), (d) pH = 9 (buffer: 10 mM Gly), (e) pH = 10 (buffer: 10 mM Gly). (black square) 0 M NaCl, (white square) 0.01 M NaCl, (black circle) 0.02 M NaCl, (white circle) 0.05 M NaCl, (black up-triangle) 0.1 M NaCl, (white up-triangle) 0.2 M NaCl, (black down-triangle) 0.5 M NaCl, (white down-triangle) 1.0 M NaCl. The concentration (*P*_t_) of Ssh10b was 50 μM.

**Figure 3:**
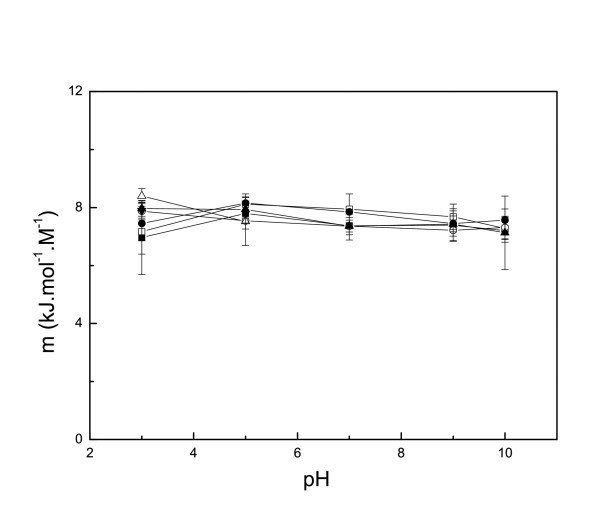
**The m value *v.s*. pH**. (black square) 0 M NaCl, (white square) 0.01 M NaCl, (black circle) 0.02 M NaCl, (white circle) 0.05 M NaCl, (black up-triangle) 0.1 M NaCl, (white up-triangle) 0.2 M NaCl. The experimental condition is in Figure 3.

**Figure 4:**
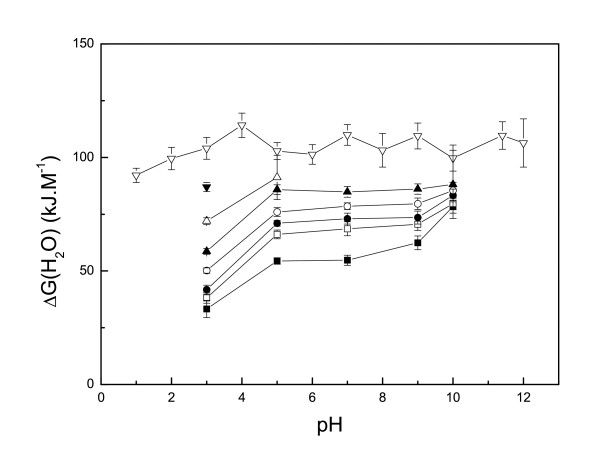
**The pH dependence of ΔG(H_2_O) of Ssh10b of urea denaturation at different NaCl concentrations**. (black square) 0 M NaCl, (white square) 0.01 M NaCl, (black circle) 0.02 M NaCl, (white circle) 0.05 M NaCl, (black up-triangle) 0.1 M NaCl, (white up-triangle) 0.2 M NaCl, (black down-triangle) 0.5 M NaCl. The experimental condition is in Figure 3. The pH dependence of ΔG(H_2_O) of Ssh10b of GdnHCl denaturation (white down-triangle).

Within the pH range 1 to 12, the CD spectra of native Ssh10b in high ionic strength phosphate buffer (20 mM Na_2_HPO_4_/NaH_2_PO_4_) [16] were nearly identical with just a slight difference below 205 nm, indicating that the protein was stable over this pH range. Ssh10b was resistant to urea-induced denaturation in phosphate buffer (20 mM Na_2_HPO_4_/NaH_2_PO_4_) [16], but showed pH- and salt-dependent stability toward urea denaturation in the monovalent ion buffer. At 25°C, we analyzed urea-induced unfolding at five different pH values (3, 5, 7, 9 and 10) and at eight different salt concentrations (0 M, 0.01 M, 0.02 M, 0.05 M, 0.1 M, 0.2 M, 0.5 M and 1 M NaCl). At low NaCl concentrations (<0.2 M) the CD signal at 220 nm showed a two-state cooperative unfolding transition, while at high salt concentrations (>0.2 M NaCl) Ssh10b was resistant to urea-induced denaturation. Figure 1 shows a typical profile of the ellipticity as function of urea concentration. At pH <3, the protein was slightly unfolded, while at pH >11, the urea-unfolded protein irreversibly aggregated (Figure 2). Fitting the curves separately yields m values of around 7.5 ± 1 kJ mol^-1^M^-1 ^(Figure 3). The unfolding free energies, ΔG(H_2_O), were obtained by fitting the transition curves to equation 5, as shown in Figure 4 and summarized in Table 1. The protein stability increased with increasing pH and salt concentration. In the absence of NaCl, the unfolding ΔG of the protein increased from about 33 kJ/mol at pH 3 to about 78 kJ/mol at pH 10. At all values of pH used, the ΔG was observed to increase with increasing NaCl concentration. At pH 3, the unfolding ΔG of the protein increased from about 33 kJ/mol in the absence of NaCl to about 87 kJ/mol in the presence of 0.5 M NaCl; while at pH 10, the increase was from about 78 kJ/mol in the absence of NaCl to about 88 kJ/mol in the presence of 0.1 M NaCl (at higher NaCl concentrations, the protein is resistant to denaturation by urea), implying that increasing pH or NaCl concentration have similar effects on the protein stability.

**Table 1:** Thermodynamic parameters derived from urea denaturation at different concentrations of NaCl

	pH 3			pH 5			pH 7			pH 9			pH 10		
NaCl	ΔG(H_2_O)	m	C_m_	ΔG(H_2_O)	m	C_m_	ΔG(H_2_O)	m	C_m_	ΔG(H_2_O)	m	C_m_	ΔG(H_2_O)	m	C_m_
(M)	(kJ.mol^-1^)	(kJ.mol^-1^.M^-1^)	(M)	(kJ.mol^-1^)	(kJ.mol^-1^.M^-1^)	(M)	(kJ.mol^-1^)	(kJ.mol^-1^.M^-1^)	(M)	(kJ.mol^-1^)	(kJ.mol^-1^.M^-1^)	(M)	(kJ.mol^-1^)	(kJ.mol^-1^.M^-1^)	(M)

0	33.3	7.0	1.3	54.4	7.8	3.8	54.7	7.4	4.1	62.4	7.4	5.1	78.3	7.2	7.5
0.01	38.2	7.2	1.9	66.1	8.1	5.1	68.6	7.9	5.5	70.6	7.7	6.0	79.7	7.3	7.6
0.02	41.8	7.4	2.3	71.0	8.2	5.7	73.1	7.8	6.2	73.5	7.4	6.6	83.3	7.6	7.8
0.05	50.2	7.9	3.3	75.9	7.5	6.8	78.5	7.4	7.3	79.6	7.2	7.6	85.5	7.3	8.3
0.1	58.6	8.0	4.3	85.8	7.9	7.7	84.8	7.4	8.2	86.1	7.4	8.3	88.1	7.1	8.9
0.2	72.0	8.4	5.6	91.2	7.5	8.9									
0.5	87.0	7.9	7.9												
1															

### Acid/base titration of native Ssh10b monitored by potentiometry

**Figure 5:**
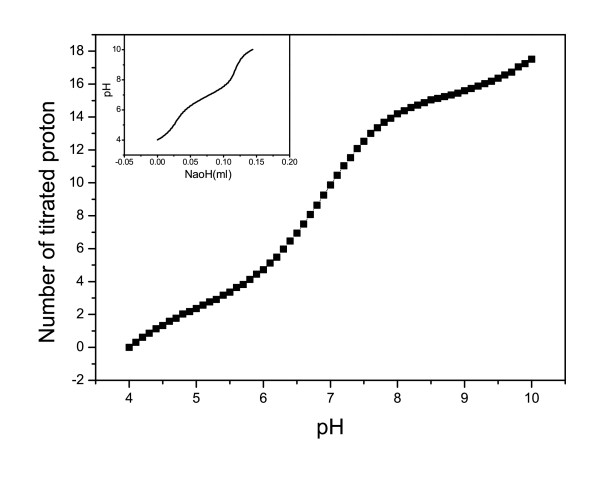
**H^+ ^titration curve of the Ssh10b protein**. The inset shows the original experimental curve. A 12 mL portion of protein solution at a concentration of approximately 0.95 mg/mL was used. Blanks consisted of 12 mL of water. The titration was carried out immediately after adjusting the pH to 4.0 using microliter amounts of 0.1575 M HCl, measured at 25°C.

Potentiometric H^+ ^titrations at 25°C are shown in Figure 5. The inset in Figure 5 shows the original titration plot. The equivalents of NaOH used in the titration were subtracted from the equivalents of NaOH used in a control experiment. The control experiment consisted of a sample of degassed water using an identical titration protocol described above, but with no protein in the sample. The Ssh10b concentration was 0.95 mg/mL. The moles of H^+ ^bound per mole of protein was calculated according to equation 7 from the titration volume, the normality of the titrant, and the number of moles (of monomer) of protein. For convenience, the number of titrated protons at pH 4.0 was arbitrarily set to zero. The results demonstrated that 18 of 27 structurally resolved protonated groups in Ssh10b (3 Asp, 5 Glu, 8 Lys, 8 Arg, 1 Tyr, and N- and C- termini) were neutralized over the pH range 4–10. Since Ssh10b contains 1 Tyr and no His or Cys residues, the presence of 18 titratable residues in the pH range 4–10 indicates that the pKa values of the ionizable residues are different in the native and unfolded proteins [11].

### pH-dependent unfolding by GdnHCl in monovalent ionic buffer

At pH 3, when the concentration of NaCl is greater than 0.5 M, the protein was resistant to denaturation by urea. Due to the limitation of the urea solubility, determining the unfolding ΔG at high salt concentration was not possible. For comparison, we also analyzed GdnHCl-induced unfolding in the pH range 1 to 12 in monovalent ion buffer. Upon incubation in 6 M GdnHCl for 24 h at 25°C, Ssh10b showed a significant reduction in backbone CD signal. The data were fitted to a two-state model (equations 3 to 6) and the pH value, free energy changes for unfolding (ΔG(H_2_O)), slope (m), and mid-point for GdnHCl denaturation (Cm) are summarized in Table 2. The results show that the denaturant m value increases slightly with increasing pH, from 16.3 at pH 1 to 20.0 at pH 6, and then the m value decreases slightly with further increase in pH, to about 18 at pH 12. The m value is relatively constant with changing pH, suggesting that there is no obvious population of an intermediate during unfolding [17]. That the m value for GdnHCl denaturation is about double that for urea denaturation is in agreement with general observation [18]. It is interesting to notice that the m value for GdnHCl denaturation is also buffer-dependent. The m value for GdnHCl denaturation is about 23 kJ mol^-1^M^-1 ^in phosphate buffer [16], about 6.6 kJ mol^-1^M^-1 ^in MOPS buffer [19], and about 20 kJ mol^-1^M^-1 ^in HEPES buffer (this work).

The unfolding free energies, ΔG(H_2_O), are shown in Figure 3. It is clear that the value of ΔG(H_2_O) fluctuated slightly with changes in the pH. A maximum stability of Ssh10b of about 110 kJ/mol was observed in the pH range 4–8 at 25°C, which is only about 15 kJ/mol higher than that at low (or high) pH, where the electrostatic interactions of the ion-pairs are disrupted. The observation of small changes in the thermodynamic parameters measured by GdnHCl denaturation in the pH range 1–12 suggests that the ionization state of the residues had little effect on the stability of the Ssh10b protein.

**Table 2:** Thermodynamic parameters derived from GdnHCl denaturation for the conformational stability of Ssh10b at 25°C. The protein concentration was 50 μm

**pH**	**ΔG(H**_2_**O) (kJ.mol**^-1^**)**	**m (kJ.mol**^-1^**.M**^-1^**)**	**C**_m_**(M)**
1	94.7	16.3	4.25
2	95.9	16.3	4.37
3	107.8	18.6	4.48
4	108.5	18.4	4.56
5	112.7	19.6	4.51
6	111.4	20.0	4.34
7	100.9	17.7	4.30
8	101.4	17.7	4.30
9	99.8	17.8	4.24
10	99.5	17.3	4.29
11.4	108.7	18.8	4.46
12	105.0	17.9	4.49

## Discussion

The crystal structure of Ssh10b reveals that the monomer of the protein consists of 27 ionizable residues (3 Asp, 5 Glu, 8 Lys, 8 Arg, 1 Tyr, and N and C termini), all of which are surface-exposed, except the two residues Tyr22 and Glu54; there are salt bridges involving Glu36-Lys68, Glu54-Arg57, Asp63-Lys97, and Glu66-Arg95 and an ion-pair network involving Lys40-Glu91-Arg71-Glu69 [15]. The number of ionizable residues is clearly higher than the average value, which is around five ion-pairs per 150 residues of protein [20].

At pH <11 (the isoelectric point) [16], Ssh10b has net positive charges on the protein surface. The charge-charge interactions between the positive charges are repulsive and unfavorable for the folded protein. The protein should adopt a structure that will optimize the charge-charge interactions and reduce the repulsive forces. Since Ssh10b contains only 1 Tyr and no His or Cys residues, the presence of 18 titratable residues in the dimeric protein in the pH range 4–10 indicates that the pKa values of the ionizable residues are different between the native and unfolded protein with the pKa values downshifted for the alkaline residues Lys and Arg [11]. In the pH range 4–10, with the increase of pH, the alkaline residues Lys and Arg with the downshifted pKa values could be gradually neutralized, resulting in a decrease in the net positive charge and enhancement of the protein stability. This is consist with the experimental observation: in the absence of NaCl and in low ionic strength buffer, the stability of Ssh10b exhibited a strong pH dependence, and the unfolding ΔG of the protein increased from about 33 kJ/mol at pH 3 to about 78 kJ/mol at pH 10, showing that the increased number of charged residues in thermophilic Ssh10b does not contribute to the increased stability of the folded protein.

It has long been understood that salt has a significant impact on the stability of proteins by altering the interactions of the aqueous solvent with the protein through preferential hydration, called the Hofmeister effect, as well as by screening electrostatic interactions between charged residues on the protein surface [7,21]. Since the interactions between charges of Ssh10b are predominantly repulsive at pH 4–10, then salt will have a stronger screening effect on these interactions and will serve to stabilize the folded protein (the salting-out effect), agreeing well with the experimental observation that the unfolding ΔG increased with increasing salt concentration.

Urea and GdnHCl denaturation curves are generally used to obtain an estimate of the protein free energy. The reasons for differences in the parameters obtained by urea and GdnHCl-induced denaturation have been extensively discussed [22-26]. It is well understood that GdnHCl is a salt and, therefore it is expected to ionize in aqueous solution, while urea is not. The Gdn^+ ^and Cl^- ^ions could mask the charged residues and screen electrostatic interactions between charged residues on the protein surface. If the charge-charge interactions are repulsive, the unfolding ΔG obtained by urea-induced denaturation should be lower than that obtained by GdnHCl-induced denaturation; while if the interactions are attractive, the unfolding ΔG obtained by urea-induced denaturation should be higher than that obtained by GdnHCl-induced denaturation. We observed that the unfolding ΔG obtained by urea-induced denaturation is smaller than that obtained by GdnHCl-induced denaturation. In addition, the value obtained by GdnHCl-induced denaturation in HEPES buffer (this work) is smaller than that in phosphate buffer (about 123 kJ mol^-1^, in 20 mM Na_2_HPO_4_/NaH_2_PO_4_) [16], which also supports the suggestion that the increased number of charged residues in thermophilic Ssh10b does not contribute to the increased stability of the folded protein.

Our results indicate that the ionizable residues and ion-pairs probably do not play a major role in stability of the native Ssh10b protein. What is their role? Several recent studies have shown that charge-charge interactions influence the denatured state ensemble and contribute to protein stability [27,28]. When both the hydrophobic and hydrogen-bonding interactions that stabilize the folded state are disrupted, the unfolded polypeptide chain rearranges to more compact conformations with favorable long-range electrostatic interactions. In general, a compact denatured ensemble results in a decrease in the change in solvent-accessible area, ΔASA, between the native and the unfolded proteins. Across many protein folding systems, there is a correlation between the m value and ΔASA, as well as between the number of residue and ΔASA [18]. The Ssh10b dimer consists of 194 amino acid residues. This predicts a ΔASA value of 17135 and an m value of about 9 kJ mol^-1^M^-1 ^for urea denaturation, which is appreciably larger than that determined experimentally (7.5 ± 1 kJ mol^-1^M^-1^). Tanford showed that the m value depends on the groups in a protein that are buried in the native state but exposed to solvent in the denatured state [23]. That the experimentally determined m value is smaller than the theoretically predicted value suggests a denatured protein with a compact ensemble. This is consistent with the results in previous study. It was found that the value of the heat capacity change, Δ*C*_p_, of Ssh10b unfolding with the value of 19 J T^-1 ^mol residue^-1 ^is significantly smaller than that of the average value for proteins from mesophiles (50 J T^-1 ^mol residue^-1^) or the value calculated from the Ssh10b structural data (64 J T^-1 ^mol residue^-1^), suggesting a denatured protein with compact ensemble [16].

## Conclusion

These findings suggest that the increased number of charged residues and ion pairs in protein Ssh10b from hyperthermophiles does not contribute to the increased stability of the folded protein, but may affect the denatured state ensemble leading to the enhanced denaturation temperature.

## Methods

### Materials

Urea, GdnHCl, Gly, HEPES, HEPES sodium salt, MES hydrate, MES sodium salt, ethanolamine hydrochloride, and ethanolamine were purchased from Sigma. Isopropyl β-D-thio-galactoside (IPTG) was purchased from Merck, and all other reagents were local products of analytical grade. Twice-deionized water was used throughout.

### Protein purification

The Ssh10b gene was provided by Professor J. F. Wang from the Institute of Biophysics, Chinese Academy of Sciences, and Professor L. Huang from the State Key Laboratory of Microbial Resources, Institute of Microbiology, Chinese Academy of Sciences[14] The Ssh10b protein was produced in *E. coli *and purified as previously described[16] The protein samples were dialyzed against water then freeze-dried and stored at -20°C.

### Buffer preparation

The buffers used for the urea denaturation experiments were 10 mM Gly (pH 3, 9, 10), 10 mM HAc (pH 5), and 10 mM HEPES (pH 7). A set of protein samples with urea concentrations ranging from 0 to 10 M were prepared by mixing appropriate amounts of protein stock and two solutions containing either 0 or 10 M urea in 0 M, 0.01 M, 0.02 M, 0.05 M, 0.1 M, 0.2 M, 0.5 M or 1.0 M NaCl. The concentrations of the urea stock solutions were determined by measurement of the refractive index. Urea solutions were prepared fresh on the day of use.

The following buffers were used for the GdnHCl denaturation experiments at concentrations of 50 mM, except for HCl at 100 mM, to cover the pH range specified: HCl (pH 1.0), H_3_PO_4_-KH_2_PO_4 _(pH 2.0–3.0), HAc-NaAc (pH 4.0–5.0), MES hydrate-MES sodium salt (pH 6.0), HEPES sodium salt-HEPES (pH 7.0–8.0), ethanolamine hydrochloride-ethanolamine (pH 9.0–10.0), K_2_HPO_4_.3H_2_O-K_3_PO_4_.7H_2_O (pH 11.4–12.0).

### Unfolding studies

The unfolding of Ssh10b by urea and GdnHCl was monitored by circular dichroism (CD) using a Jasco-J720 Spectropolarimeter. The CD signal in the region 190–250 nm (far UV CD) was monitored using a rectangular quartz cuvette with a path length of 1 mm. The samples containing 50 μM protein and various concentrations of urea (or GdnHCl) were equilibrated for 24 h at 25°C before experiments.

### Analysis of the denaturation data

The thermodynamic properties of Ssh10b were calculated assuming a two-state denaturation process, N↔K22U
 MathType@MTEF@5@5@+=feaafiart1ev1aaatCvAUfKttLearuWrP9MDH5MBPbIqV92AaeXatLxBI9gBaebbnrfifHhDYfgasaacPC6xNi=xH8viVGI8Gi=hEeeu0xXdbba9frFj0xb9qqpG0dXdb9aspeI8k8fiI+fsY=rqGqVepae9pg0db9vqaiVgFr0xfr=xfr=xc9adbaqaaeGacaGaaiaabeqaaeqabiWaaaGcbaGaeeOta40aaSraaSqaaiabikdaYaqabaGcdaGd4aWcbeqaaiabdUealbGccaGLugcacqaIYaGmcqqGvbqvaaa@330F@. Concentrations of the folded protein [N_2_] (in dimer units) and the unfolded protein [U] (in monomer units) at different temperatures or denaturant concentrations were calculated according to [16,29]:

[N2]=Pt/2×y−yU+mUT[D]yN+mNT[D]−yU+mUT[D]
 MathType@MTEF@5@5@+=feaafiart1ev1aaatCvAUfKttLearuWrP9MDH5MBPbIqV92AaeXatLxBI9gBaebbnrfifHhDYfgasaacPC6xNi=xI8qiVKYPFjYdHaVhbbf9v8qqaqFr0xc9vqFj0dXdbba91qpepeI8k8fiI+fsY=rqGqVepae9pg0db9vqaiVgFr0xfr=xfr=xc9adbaqaaeGacaGaaiaabeqaaeqabiWaaaGcbaGaei4waSLaemOta40aaSbaaSqaaiabikdaYaqabaGccqGGDbqxcqGH9aqpcqWGqbaudaWgaaWcbaGaeeiDaqhabeaakiabc+caViabikdaYiabgEna0MqbaoaalaaabaGaemyEaKNaeyOeI0IaemyEaK3aaSbaaeaacqqGvbqvaeqaaiabgUcaRiabd2gaTnaaBaaabaGaeeyvaufabeaacqWGubavcqGGBbWwcqqGebarcqGGDbqxaeaacqWG5bqEdaWgaaqaaiabb6eaobqabaGaey4kaSIaemyBa02aaSbaaeaacqqGobGtaeqaaiabdsfaujabcUfaBjabbseaejabc2faDjabgkHiTiabdMha5naaBaaabaGaeeyvaufabeaacqGHRaWkcqWGTbqBdaWgaaqaaiabbwfavbqabaGaemivaqLaei4waSLaeeiraqKaeiyxa0faaaaa@5DF4@

[U]=PtyN+mNT[D]−yyN+mNT[D]−yU+mUT[D]
 MathType@MTEF@5@5@+=feaafiart1ev1aaatCvAUfKttLearuWrP9MDH5MBPbIqV92AaeXatLxBI9gBaebbnrfifHhDYfgasaacPC6xNi=xI8qiVKYPFjYdHaVhbbf9v8qqaqFr0xc9vqFj0dXdbba91qpepeI8k8fiI+fsY=rqGqVepae9pg0db9vqaiVgFr0xfr=xfr=xc9adbaqaaeGacaGaaiaabeqaaeqabiWaaaGcbaGaei4waSLaemyvauLaeiyxa0Laeyypa0Jaemiuaa1aaSbaaSqaaiabdsha0bqabaqcfa4aaSaaaeaacqWG5bqEdaWgaaqaaiabb6eaobqabaGaey4kaSIaemyBa02aaSbaaeaacqqGobGtaeqaaiabdsfaujabcUfaBjabbseaejabc2faDjabgkHiTiabdMha5bqaaiabdMha5naaBaaabaGaeeOta4eabeaacqGHRaWkcqWGTbqBdaWgaaqaaiabb6eaobqabaGaemivaqLaei4waSLaemiraqKaeiyxa0LaeyOeI0IaemyEaK3aaSbaaeaacqqGvbqvaeqaaiabgUcaRiabd2gaTnaaBaaabaGaeeyvaufabeaacqWGubavcqGGBbWwcqqGebarcqGGDbqxaaaaaa@58C9@

where *P*_t _is the total protein concentration in monomer units, *y *is the experimentally measured value at a given temperature *T *or given denaturant concentration [D], *y*_N _and *y*_U _are the intercepts, and *m*_N _and *m*_U _are the slopes of the native and unfolded baselines, respectively. The apparent equilibrium constant (*K*) and the corresponding free energy change (ΔG) at temperature *T *or denaturant concentration [D] could be calculated according to:

K=2×PtyN+mNT[D]−yU+mUT[D]×[yN+mNT[D]−y]2y−yU+mUT[D]
 MathType@MTEF@5@5@+=feaagaart1ev2aaatCvAUfKttLearuWrP9MDH5MBPbIqV92AaeXatLxBI9gBaebbnrfifHhDYfgasaacPC6xNi=xI8qiVKYPFjYdHaVhbbf9v8qqaqFr0xc9vqFj0dXdbba91qpepeI8k8fiI+fsY=rqGqVepae9pg0db9vqaiVgFr0xfr=xfr=xc9adbaqaaeGacaGaaiaabeqaaeqabiWaaaGcbaGaem4saSKaeyypa0tcfa4aaSaaaeaacqaIYaGmcqGHxdaTcqWGqbaudaWgaaqaaiabbsha0bqabaaabaGaemyEaK3aaSbaaeaacqqGobGtaeqaaiabgUcaRiabd2gaTnaaBaaabaGaeeOta4eabeaacqWGubavcqGGBbWwcqqGebarcqGGDbqxcqGHsislcqWG5bqEdaWgaaqaaiabbwfavbqabaGaey4kaSIaemyBa02aaSbaaeaacqqGvbqvaeqaaiabdsfaujabcUfaBjabbseaejabc2faDbaakiabgEna0MqbaoaalaaabaWaamWaaeaacqWG5bqEdaWgaaqaaiabb6eaobqabaGaey4kaSIaemyBa02aaSbaaeaacqqGobGtaeqaaiabdsfaujabcUfaBjabbseaejabc2faDjabgkHiTiabdMha5bGaay5waiaaw2faamaaCaaabeqaaiabikdaYaaaaeaacqWG5bqEcqGHsislcqWG5bqEdaWgaaqaaiabbwfavbqabaGaey4kaSIaemyBa02aaSbaaeaacqqGvbqvaeqaaiabdsfaujabcUfaBjabbseaejabc2faDbaaaaa@6C80@

Δ*G *= -*RT *ln *K*

where *R *is the gas constant and *T *is the absolute temperature. According to the linear free energy model, the changes in free energy, enthalpy, and entropy that occur on unfolding are expected to vary linearly with the denaturant concentration [D]:

Δ*G *= Δ*G*(H_2_O) - *m *[D]

where ΔG(H_2_O) represents the free energy change of unfolding in the absence of denaturant and *m *is the slope of the transition for the free energy change. The midpoint of the transition (*C*_m_), where 50% of the protein is unfolded, is a function of protein concentration *P*_*t*_

Cm=Cm0+RTln⁡(Pt)m
 MathType@MTEF@5@5@+=feaafiart1ev1aaatCvAUfKttLearuWrP9MDH5MBPbIqV92AaeXatLxBI9gBaebbnrfifHhDYfgasaacPC6xNi=xI8qiVKYPFjYdHaVhbbf9v8qqaqFr0xc9vqFj0dXdbba91qpepeI8k8fiI+fsY=rqGqVepae9pg0db9vqaiVgFr0xfr=xfr=xc9adbaqaaeGacaGaaiaabeqaaeqabiWaaaGcbaGaem4qam0aaSbaaSqaaiabd2gaTbqabaGccqGH9aqpcqWGdbWqdaqhaaWcbaGaemyBa0gabaGaeGimaadaaOGaey4kaSscfa4aaSaaaeaacqWGsbGucqWGubavcyGGSbaBcqGGUbGBcqGGOaakcqWGqbaudaWgaaqaaiabbsha0bqabaGaeiykaKcabaGaemyBa0gaaaaa@3FD8@

where Cm0=ΔG(H2O)m
 MathType@MTEF@5@5@+=feaafiart1ev1aaatCvAUfKttLearuWrP9MDH5MBPbIqV92AaeXatLxBI9gBaebbnrfifHhDYfgasaacPC6xNi=xI8qiVKYPFjYdHaVhbbf9v8qqaqFr0xc9vqFj0dXdbba91qpepeI8k8fiI+fsY=rqGqVepae9pg0db9vqaiVgFr0xfr=xfr=xc9adbaqaaeGacaGaaiaabeqaaeqabiWaaaGcbaGaem4qam0aa0baaSqaaiabd2gaTbqaaiabicdaWaaakiabg2da9KqbaoaalaaabaGaeuiLdqKaem4raCKaeeikaGIaeeisaG0aaSbaaeaacqqGYaGmaeqaaiabb+eapjabbMcaPaqaaiabd2gaTbaaaaa@3A34@ is the transition midpoint at protein concentration *P*_t _= 1 M.

### Acid/base titration of native Ssh10b monitored by potentiometry

Measurements under native conditions were done with a Titrando 809 pH/ion meter (Metrohm, LTD, Switzerland) equipped with a Solitrode electrode and an LL reference system. The electrode was calibrated immediately prior to experiments using a set of certified standard buffer solutions at pH 4.00, 7.00, and 9.00. The pH response was linear over the calibrated pH range, and the readings were stable and reproducible. All samples dissolved in deionized water were extensively degassed. After an initial and extensive dialysis at 4°C, samples were centrifuged in a benchtop centrifuge, and then dialyzed overnight in a CO_2_-free atmosphere against water at 4°C. A 12 mL portion of protein solution at a concentration of approximately 0.95 mg/mL was used. Blanks consisted of 12 mL of water. The titration was carried out immediately after adjusting the pH to 4.0 using microliter amounts of 0.1575 M HCl. The Ssh10b sample solution was continuously titrated from pH 4.00 to pH 10.00 with aliquots (1 μL) of 0.1551 M NaOH, and the pH values and volumes added were recorded. Multiple titration curves were always measured, and reversibility was checked routinely.

A proton titration curve represents the number of moles of protons bound or released per mole of protein as the pH is changed. The number of protons that reacted that were derived from the protein is calculated from the difference between the titration of the protein solution and that of a blank water sample. The total concentration of OH^- ^in solution as titrant was added was calculated from the following:

[OH^-^]_total _= Δ[OH^-^] - Δ[H^+^] + [H^+^]_pro_,

where Δ[OH^-^] and Δ[H^+^] represent the changes in [OH^-^] and [H^+^] due to the dissociation of water during the titration, respectively. [H^+^]_pro _represents the amount of H^+ ^derived from the protein sample. Δ[H^+^] is predominant in the acidic range of pH, and Δ[OH^-^] is predominant in the basic range of pH. The number of titrated protons was then calculated from the proton titration curve. Since the measurements were performed at low protein concentration (i.e. about 1 mg/mL or 0.1 mM), and the protein was a classic polyampholyte with both weak acidic and basic groups, most of the dissociated residues could be detected in the pH range of 4.00 to 10.00 as shown in the results.

## Authors' contributions

YM carried out the experimental work and participated in the data analysis of the study. XS participated in drafting the manuscript. XP conceived this study and wrote the manuscript. All authors read and approved the final manuscript.
